# Duct- and Acinar-Derived Pancreatic Ductal Adenocarcinomas Show Distinct Tumor Progression and Marker Expression

**DOI:** 10.1016/j.celrep.2017.09.093

**Published:** 2017-10-24

**Authors:** Rute M.M. Ferreira, Rocio Sancho, Hendrik A. Messal, Emma Nye, Bradley Spencer-Dene, Richard K. Stone, Gordon Stamp, Ian Rosewell, Alberto Quaglia, Axel Behrens

**Affiliations:** 1 Adult Stem Cell Laboratory, The Francis Crick Institute, 1 Midland Road, London NW1 1AT, UK; 2 Experimental Histopathology, The Francis Crick Institute, 1 Midland Road, London NW1 1AT, UK; 3 Transgenic Service–Biological Research Facility, The Francis Crick Institute, 1 Midland Road, London NW1 1AT, UK; 4 King’s College Hospital/King’s College London, Institute of Liver Studies, Denmark Hill, London SE5 9RS, UK; 5 King’s College London, Faculty of Life Sciences and Medicine, Guy’s Campus, London SE1 1UL, UK

**Keywords:** PDAC, pancreatic cancer, mouse models, progression, PanINs, AGR2, cell of origin

## Abstract

The cell of origin of pancreatic ductal adenocarcinoma (PDAC) has been controversial. Here, we show that identical oncogenic drivers trigger PDAC originating from both ductal and acinar cells with similar histology but with distinct pathophysiology and marker expression dependent on cell of origin. Whereas acinar-derived tumors exhibited low AGR2 expression and were preceded by pancreatic intraepithelial neoplasias (PanINs), duct-derived tumors displayed high AGR2 and developed independently of a PanIN stage via non-mucinous lesions. Using orthotopic transplantation and chimera experiments, we demonstrate that PanIN-like lesions can be induced by PDAC as bystanders in adjacent healthy tissues, explaining the co-existence of mucinous and non-mucinous lesions and highlighting the need to distinguish between true precursor PanINs and PanIN-like bystander lesions. Our results suggest AGR2 as a tool to stratify PDAC according to cell of origin, highlight that not all PanIN-like lesions are precursors of PDAC, and add an alternative progression route to the current model of PDAC development.

## Highlights

• PDAC can originate from acinar or ductal cells via distinct modes of progression
• Duct-derived PDAC develops in a PanIN-independent manner
• PanIN-like lesions can occur in non-mutant tissue as tumor “bystanders”
• High AGR2 distinguishes duct-derived from acinar-derived PDAC

## Graphical Abstract

**Figure:**
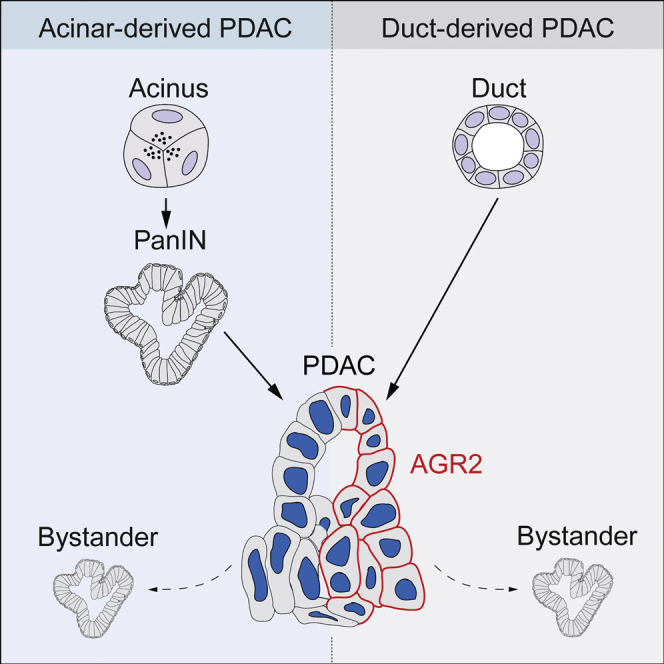

## Teaser

Pancreatic intraepithelial neoplasias (PanINs) are established precursor lesions to pancreatic ductal adenocarcinoma (PDAC). Ferreira et al. show that this model represents acinar-derived PDAC and that, in contrast, duct-derived PDAC does not develop via PanINs. PanIN-like lesions occurring alongside duct-derived tumors arise from adjacent non-mutant tissue and do not represent tumor precursors.

## Introduction

Pancreatic ductal adenocarcinoma (PDAC) remains a growing problem, despite efforts to improve diagnosis and therapy. A better understanding of early PDAC events and cellular biology are needed for improved clinical success (Ying et al., 2016).

Four types of pre-neoplastic PDAC precursors have been identified: intraductal papillary mucinous neoplasia (IPMN), pancreatic mucinous cystic neoplasm (MCN), intraductal tubular papillary neoplasm (ITPN), and pancreatic intraepithelial neoplasia (PanIN) (Cooper et al., 2013, Hruban et al., 2000a, Hruban et al., 2007). These precursors are associated with different PDAC prognoses (Cooper et al., 2013, von Figura et al., 2014). However, PanIN lesions can co-exist with other types of pre-neoplastic lesions (Brosens et al., 2015, Verbeke, 2010). This raises important issues regarding the independence and role of different lesions in progressing to PDAC. Understanding the different origins and progression of PDAC tumors will illuminate PDAC biology and could identify better markers allowing patient stratification in the clinic.

Due to late-stage diagnosis of human PDAC, genetically engineered mouse (GEM) models are the best tool to study PDAC early development. Expression of oncogenic KRas in adult mouse acinar cells, together with an inflammatory environment, can induce transdifferentiation into duct-like cells (known as acinar-to-ductal metaplasia [ADM]), formation of PanIN lesions, and culmination in PDAC (Guerra et al., 2007, Habbe et al., 2008, Kopp et al., 2012). However, human pancreatic tumor histology has previously implicated the ductal compartment in PDAC (Cubilla and Fitzgerald, 1976, Hruban et al., 2000b).

Whether pancreatic duct cells can give rise to PDAC has been controversial. Adult mouse duct cells do not develop PDAC with activation of mutant KRas only (Brembeck et al., 2003, Kopp et al., 2012, Ray et al., 2011). With additional genetic alterations, duct cells originate PDAC, but less efficiently compared to acinar cells (Bailey et al., 2016), or forming less malignant tumors (von Figura et al., 2014). Whether these differences are due to the particular oncogene combination, or an intrinsic resistance of duct cells to oncogenic transformation, is unclear.

To understand the early events of PDAC formation from different cell types, we generated GEM models combining endogenous oncogenic KRas activation (KRas^G12D^) (Hingorani et al., 2003) with inactivation of either p53 (KP mice) or the Fbw7 tumor suppressor (KF mice) induced in either adult acinar cells (using Elastase1-Cre^ERT2^) or adult duct cells (using Ck19-Cre^ER^). In both genotypes, both acinar and duct cells gave rise to PDAC, but with distinct pathophysiology. While acinar cells gave rise to PanINs that progressed to PDAC, duct-derived tumors did not develop via PanINs or other mucinous precursors, and instead developed exclusively via non-mucinous tubular lesions. Additional PanIN-like lesions were induced by PDAC tumors as “bystanders” arising from adjacent normal pancreatic tissue. Besides differences in precursor lesions, we identified AGR2 as a biomarker that discriminates acinar- and duct-derived tumors. These results describe a second major route of PDAC progression.

## Results

### *Fbw7* Embryonic Deletion in a KRas^G12D^-Driven PDAC Model Induces Duct Overproliferation and Transformation

**Figure 1:**
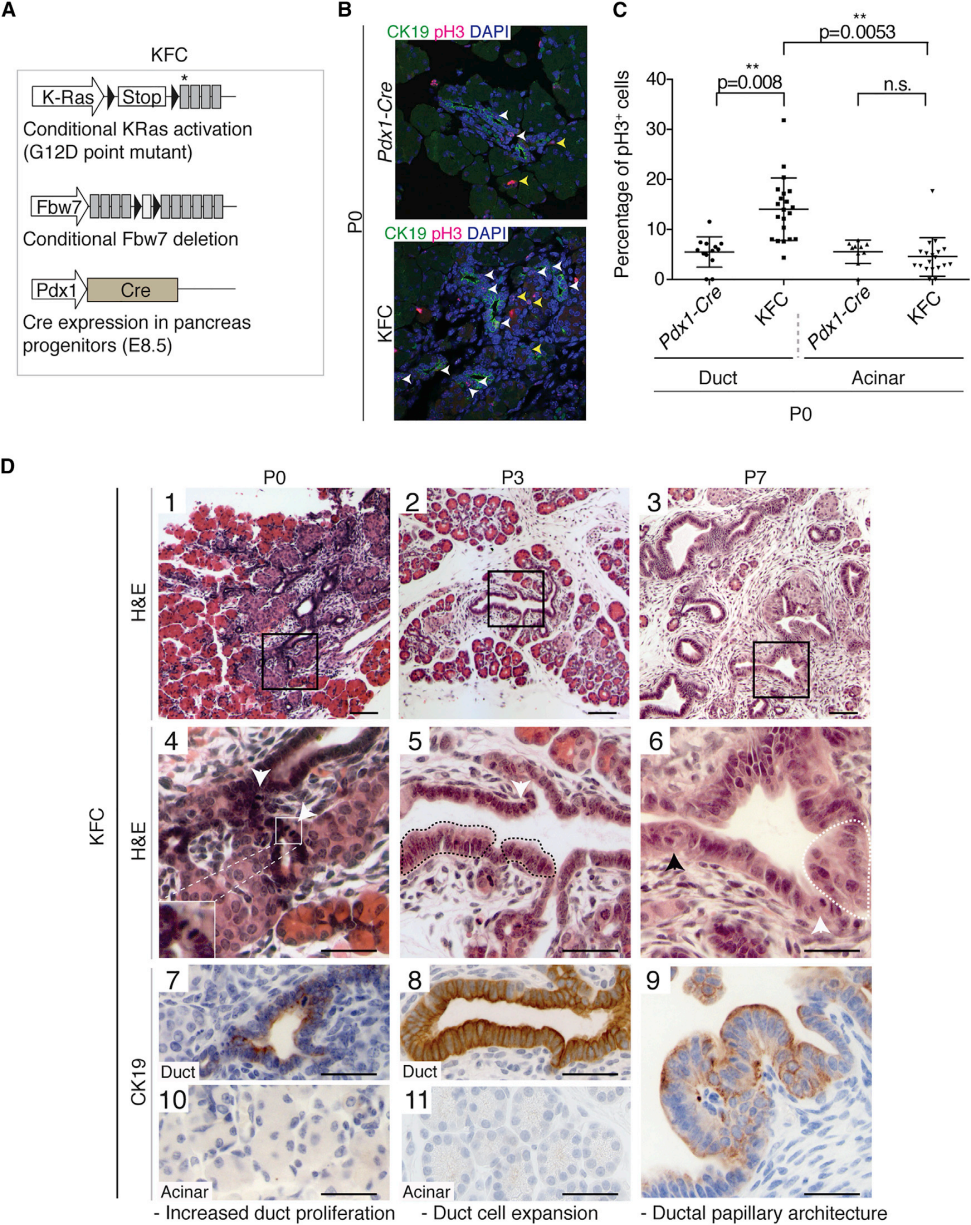
Fbw7 Embryonic Deletion Drastically Accelerates KRas^G12D^-Induced Murine PDAC and Induces an Initial Ductal Transformation (A) Schematic representation of the KFC (*KRas*^*LSL-G12D/wt*^*; Fbw7*^*F/F*^*; Pdx1-Cre*) mouse model. Cre expression is driven by the Pdx1 promoter. Black triangles indicate loxP sites. Asterisk indicates G12D point mutation. (B) Immunofluorescence staining of CK19 and pH3 in pancreas of Pdx1-Cre control and KFC mice at postnatal day 0 (P0). White arrowheads indicate pH3+ duct cells; yellow arrowheads indicate pH3+ acinar cells. (C) Quantification of pH3+ cells in acinar or ductal compartments of three P0 Pdx1-Cre and three KFC mice (as a percentage of total cells in that compartment). Mice were obtained from two independent litters. Each data point represents one pancreatic region used for quantification. Graph shows mean values ± SD. The p values were calculated using Welch’s t test (n = 3). (D) Time-course analysis of tumor development in KFC mice. (1–3) H&E low-magnification images. Black boxes indicate area magnified in images 4–6. White box highlights presence of mitotic figures in KFC ductal cells at P0. White arrows show mitotic figures. Black arrow shows loss of polarity (nuclear orientation). Black dotted lines highlight duct cell dysplasia. White dotted lines highlight pseudostratified epithelium. (7–11) CK19 IHC analysis of ductal cells (7–9) and acinar cells (10–11). For each time point, two litters, each comprising at least two KFC animals, were analyzed. Scale bars in panels 1–3 represent 100 μm. Scale bars in panels 4–11 represent 50 μm. See also Figures S1 and S2.

Fbw7 alteration has been associated with PDAC biology (Calhoun et al., 2003, Ji et al., 2015, Pérez-Mancera et al., 2012). While mutations in *FBW7* are infrequent, Fbw7 is downregulated at the protein level in PDAC patients (Ji et al., 2015). Additionally, Fbw7 acts as a tumor suppressor in a KRas^G12D^-driven PDAC embryonic model (Zhang et al., 2016). Among all pancreatic compartments, duct cells exhibit the highest levels of *Fbw7* gene expression, and *Fbw7* deletion in pancreatic progenitors by Pdx1-Cre leads to an expansion of the ductal compartment (Sancho et al., 2014), suggesting that duct cells might participate in PDAC tumorigenesis following Fbw7 loss. To test this, we induced homozygous deletion of the *Fbw7*^*F/F*^ allele in the *KRas*^*G12D*^ *Pdx1-Cre* (KC) PDAC model (Hingorani et al., 2003) to generate KFC mice (Figure 1A). As previously observed (Zhang et al., 2016), Fbw7 deletion greatly accelerated PDAC onset and markedly decreased the median survival of KFC mice compared with KC mice without changing the tumor type (Figures S1A–S1D). The percentage of mitotic cells in the ducts of adult *Fbw7*^*F/F*^*; Pdx1-Cre* (FC) mice was significantly increased compared with that in *Pdx1*-*Cre* age-matched controls, but in the acinar compartment no change in proliferation was observed (Figure S2A). In KFC mice, to avoid artifacts due to secondary effects of tumorigenesis, only very early stages of development (post-natal day 0 [P0]) were analyzed. As before, no increase in proliferation was detected in acinar cells, whereas the ductal compartment showed a marked increase in the number of mitotic cells compared with controls (Figures 1B and 1C), indicating that *Fbw7* deletion triggers overproliferation specifically in the ductal compartment.

To understand the course of PDAC development in KFC mice, we performed a detailed time course of histological analysis (Figure 1D), alongside pancreatic samples from *Pdx1-Cre* mice to distinguish oncogene-related alterations from normal postnatal changes in pancreatic morphogenesis (Shih et al., 2013) (Figure S2B). KRas oncogenic activation in the embryonic pancreas does not disturb pancreatic development, and initial transformation events are only, and rarely, detected 2 weeks after birth (Hingorani et al., 2003). In contrast, in KFC mice, Ck19-expressing low- and high-grade pre-neoplastic lesions were already evident 7 days after birth, presenting multifocal ductal structures with papillary architecture, pseudo-stratified epithelium, loss of cell polarity, and fibroinflammatory reaction (Figure 1D, 3, 6, 9).

**Figure 2:**
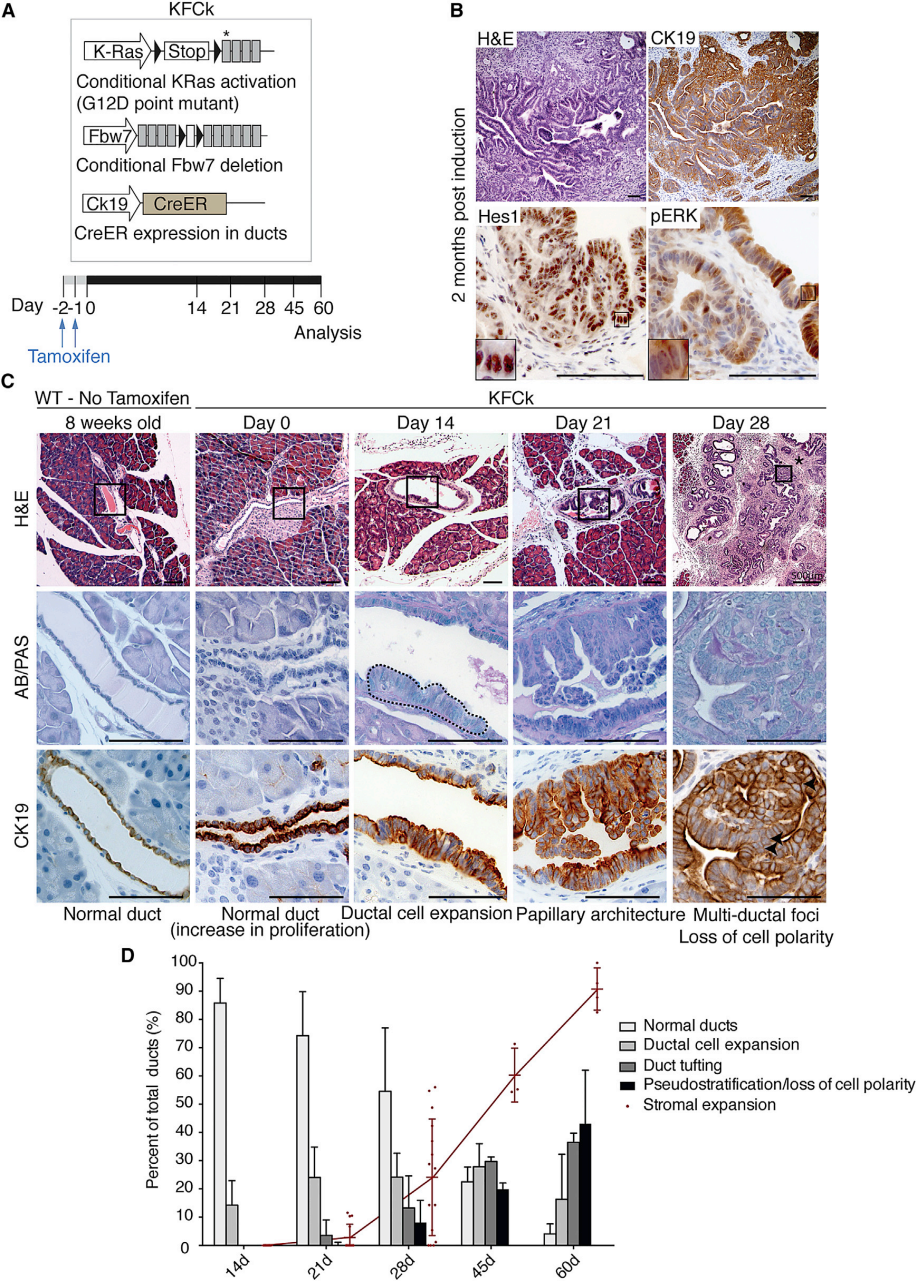
Carcinoma Initiated in Duct Cells Progresses Independently of Low-Grade PanIN Formation (A) Schematic representation of the KFCk (*KRas*^*LSL-G12D/wt*^*; Fbw7*^*F/F*^*; Ck19-Cre*^*ER*^) mouse and time-course experimental approach. Black triangles indicate loxP sites; asterisk indicates the G12D mutation. (B) Histological analysis of KFCk-derived carcinoma 2 months after tamoxifen treatment. Boxes indicate enlarged regions. (C) IHC analysis of wild-type (WT) 8-week-old pancreas and time-course analysis of carcinoma development in the KFCk model. Regions of interest marked in low-magnification H&E images are analyzed for AB/PAS staining and Ck19 immunological staining below. Black dashed line highlights ductal dysplasia. Black arrowheads indicate loss of cell polarity. At least seven animals per time point were used, and four tissue sections per animal were obtained for H&E and immunostaining. (D) Quantification of the histological progression of duct-derived tumors from the KFCk model. At least three mice per time point and three tissue sections per mouse were used. Ducts exhibiting dysplasia, tufting, or loss of polarity were counted in one category only. Bars indicate mean + SD. The percentage of ducts with stromal expansion (stromal layer thicker than 50 μm) was quantified separately (line graph shows mean ± SD). Data points indicate individual tissue sections. All scale bars represent 100 μm. See also Figure S3.

Although pancreata of newborn (P0) KFC mice (Figure 1D, 1) had a composition and architecture similar to that of controls (Figure S2B, 1), the ducts were already hyperplastic, with frequent mitotic figures (Figure 1D, 4). At P3, duct cells exhibited atypia, with increased nuclear/cytoplasmic ratio (Figure 1D, 2, 5). Acinar cells from KFC mice did not show any obvious Ck19 expression at P0 and P3 (Figure 1D, 10, 11), suggesting absence of ADM before the onset of ductal atypia. Ductal transformation in the KFC model preceded the formation of dysplastic lesions, which were detected for the first time at P7 (Figures S2C and S2D).

### Both Duct and Acinar Cells in the Adult Give Rise to PDAC but with Different Pathophysiology

Given the duct cell atypia observed in the KFC model, we generated a conditional model where *Fbw7* deletion and simultaneous KRas^G12D^ activation could be induced specifically in adult duct cells using Ck19-Cre^ER^ (KFCk mice, Figure 2A). KFCk mice induced at 8 weeks developed carcinoma approximately 2 months after oncogene activation. KFCk tumors exhibited dysplasia of ductal epithelium with tufting, and positive staining for HES1 and pERK (Figure 2B), as described for human and murine PDAC (Bardeesy et al., 2006, Hingorani et al., 2003).

At the time of oncogene activation (day 0), no alteration was detected in KFCk pancreas compared with wild-type controls, except a possible crowding of the ducts (Figure 2C). At day 14, although the acinar compartment remained morphologically unchanged, ducts became noticeably hyperplastic and 15% exhibited a duct cell expansion toward the lumen. At day 21, over 20% of ducts showed cell expansion and approximately 4% of the ducts showed dysplasia including tufting and shedding of cell clusters into the lumen. Loss of duct cell polarity, increasing nuclear atypia, stratification, and the presence of cribriform-like structures indicated the onset of carcinoma in situ, 1 month after induction (Figures 2C and 2D).

To strengthen our characterization of duct-derived tumors, we used a second genetic model, combining oncogenic KRas with inactivation of p53 (KPCk mice) (Figure S3A). KPCk pre-neoplastic lesions followed a similar histological progression as observed in KFCk mice, and mucinous PanIN lesions could not be detected at early stages of tumorigenesis (Figure S3B), suggesting that the ductal origin, rather than *Fbw7* deletion, may be responsible for the PanIN-independent mode of tumor progression.

**Figure 3:**
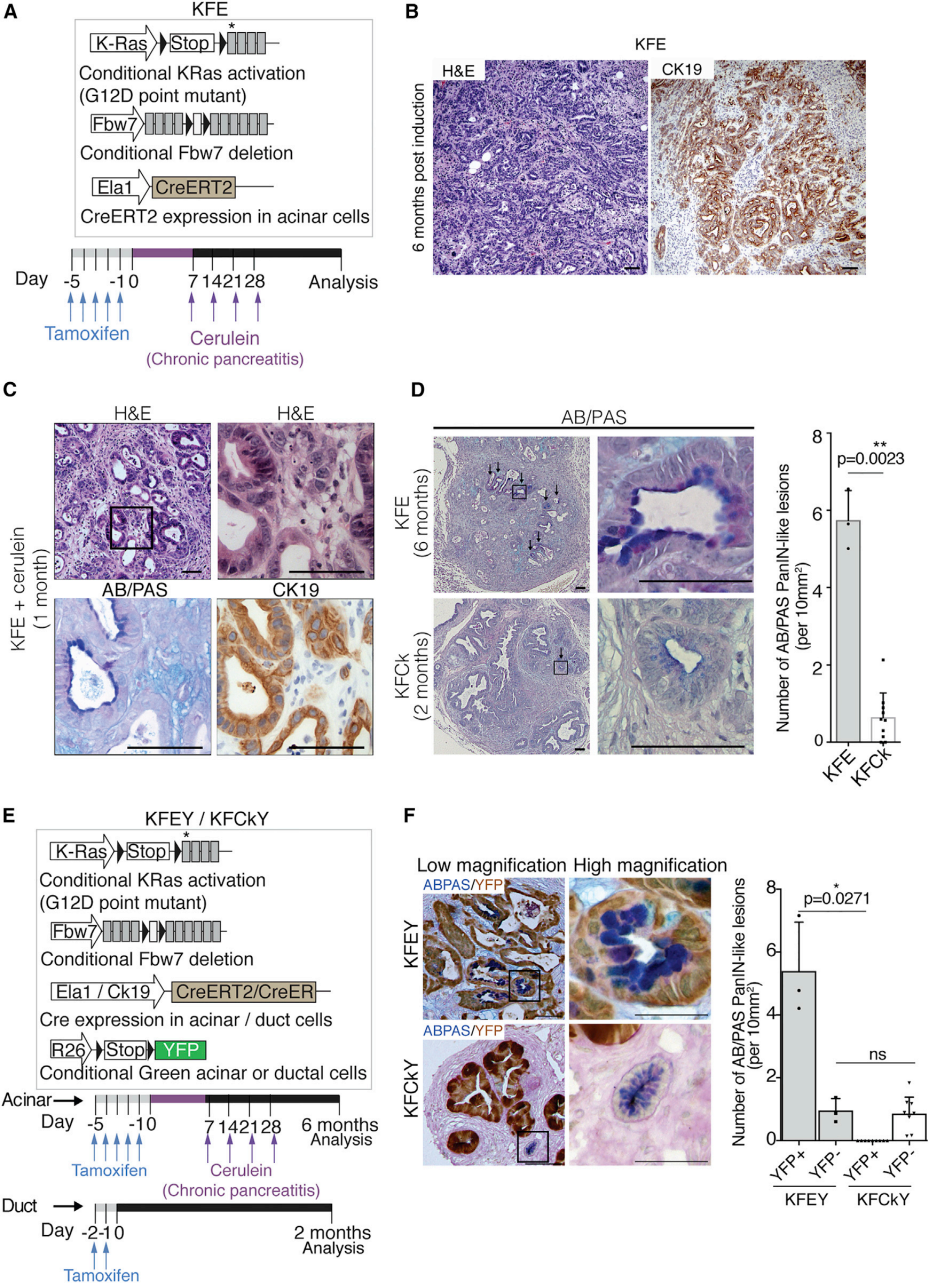
PanIN and PanIN-like Lesions Are Fundamentally Different and Occur at Different Stages of Tumor Development (A) Schematic representation of KFE (*KRas*^*LSL-G12D/wt*^*; Fbw7*^*F/F*^*; Ela1-Cre*^*ERT2*^) mice and experimental approach. Black triangles indicate loxP sites; asterisk indicates G12D mutation. (B) Histological analysis of acinar-derived tumors and corresponding CK19 IHC stain 6 months after cerulein treatment. (C) Histological analysis of KFE pancreas 1 month after cerulein treatment and corresponding CK19 and AB/PAS stain. Black box indicates region enlarged on the right. (D) AB/PAS representative images of acinar-derived (KFE) and duct-derived (KFCk) tumors, 6 and 2 months after tamoxifen treatment, respectively, and respective quantifications of AB/PAS-positive PanIN-like lesions per 10 mm^2^ of pancreatic area. Black arrows show AB/PAS-positive lesions. Black box indicates region enlarged on the right. At least three mice per genotype and three tissue sections per mouse were used. Bar chart shows mean + SD. Data points indicate scores for individual mice (n = 3 KFE mice; n = 11 KFCK mice). The p values were calculated using Welch’s t test. (E) Schematic representation of the KFEY (*KRas*^*LSL-G12D/wt*^*; Fbw7*^*F/F*^*; Ela1-Cre*^*ERT2*^*; Rosa26-LSL-YFP*) and KFCkY (*KRas*^*LSL-G12D/wt*^*; Fbw7*^*F/F*^*; Ck19-Cre*^*ER*^*; Rosa26-LSL-YFP*) mice and experimental approach. KFEY mice were treated as in Figure 3A and KFCkY mice as in Figure 2A. (F) Co-stain of YFP and AB/PAS on tumor sections from KFEY and KFCkY pancreas, 6 and 2 months after tamoxifen treatment, respectively. Quantification shows YFP-positive and -negative AB/PAS-positive PanIN-like lesions per 10 mm^2^ of pancreatic area. At least three mice per genotype and two tissue sections per mouse were used. Bar chart shows mean + SD. Data points indicate scores for individual mice (n = 3 KFEY mice; n = 10 KFCk mice). The p values were calculated using Welch’s t test. Scale bars represent 50 μm in (F) and 100 μm for the remaining panels.

To assess PDAC formation in acinar cells, we used Elastase 1-Cre^ER^ to induce *Fbw7* deletion and KRas^G12D^ activation (KFE model, Figure 3A). Cerulein treatment was used to induce chronic pancreatitis and promote PDAC formation from adult acinar cells (Carrière et al., 2011, Guerra et al., 2007) (Figure 3A). Six months after tamoxifen treatment, KFE animals developed PDAC characterized by the widespread growth of dysplastic Ck19-expressing ductal structures (Figure 3B). In contrast with duct-derived PDAC, lesions resembling low-grade PanINs were readily identified in KFE pancreas 1 month post-tamoxifen induction, well before the appearance of PDAC (Figure 3C). In late-stage carcinoma, although PanIN-like lesions were found in both acinar- and duct-derived tumors, acinar-derived carcinoma was associated with significantly more of these lesions (Figure 3D). Thus, even in the context of the same genetic alteration, acinar- and duct-derived tumors show clear differences in pathophysiology.

To better understand the nature and origin of PanIN-like lesions observed in these mouse models, we combined the acinar-targeted KFE and duct-targeted KFCk models with a *Rosa26-LSL-YFP* lineage tracer to distinguish recombined cells harboring oncogenic gene alterations from unrecombined wild-type cells (Figure 3E). Following tamoxifen treatment, plus cerulein for acinar-targeted models, both KFCkY and KFEY mice developed YFP-traced tumors. Interestingly, although in the KFEY model most of the PanIN-like lesions were both YFP and AB/PAS positive, with a minority not lineage-traced, in the duct-derived model (KFCkY) none of the PanIN-like lesions were lineage traced (Figure 3F). We inferred that the acinar-derived KFEY model develops predominantly “true” PanINs, which precede tumor onset and harbor the same oncogenic alterations as the tumor, whereas in the KFCkY model, PanIN-like lesions are less common, appear later, and are composed of cells that did not undergo Cre-mediated recombination of *KRas*^*G12D*^ and *Fbw7*^*F/F*^ alleles.

### PDAC Tumors Induce PanIN-like Lesions in Adjacent Wild-Type Tissue

**Figure 4:**
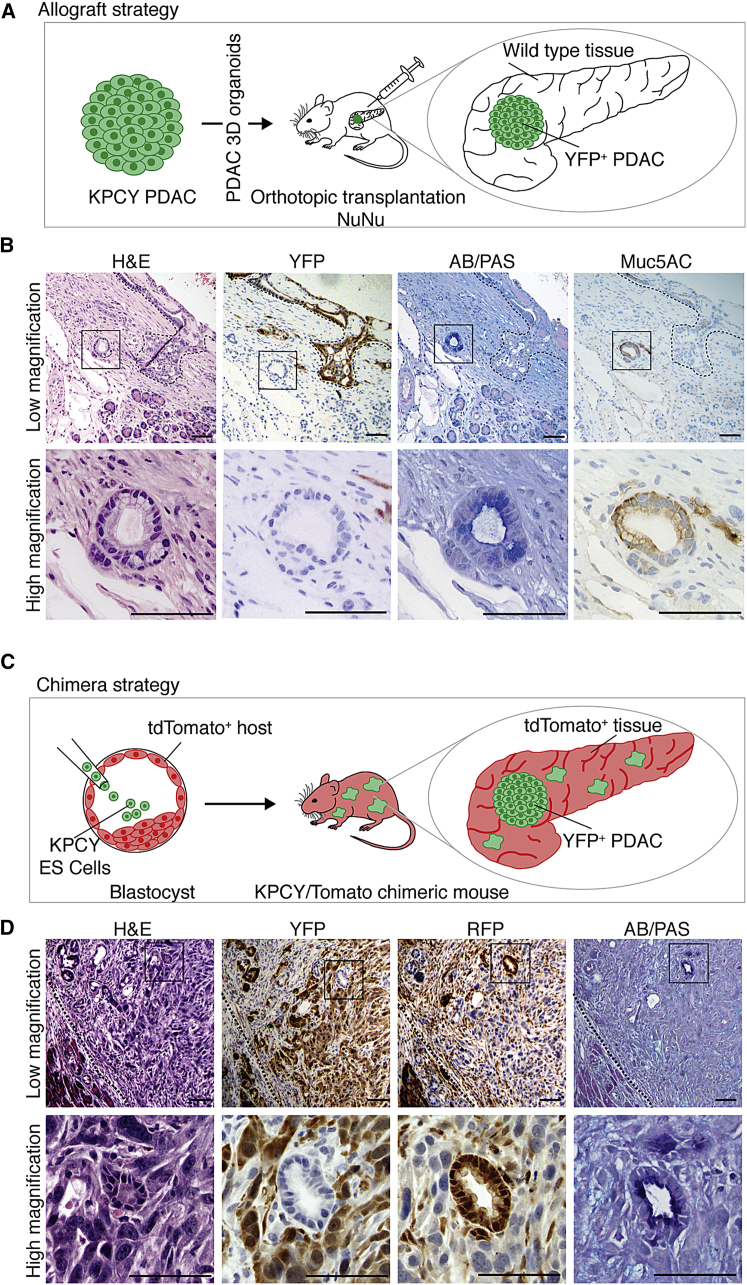
PDAC Oncogenesis Promotes Low-Grade PanIN-like Lesion Formation in Adjacent Wild-Type Tissues (A) Schematic representation of the allograft experimental approach. PDAC organoids from 5-week-old KPCY (*KRas*^*LSL-G12D/wt*^*; p53*^*F/F*^*; Pdx1-Cre; Rosa26-LSL-YFP*) mice were sorted for YFP before transplantation. (B) Histological analysis of orthotopic allograft tumors 20 days post-transplantation on serial sections. Two independent procedures were performed with four mice each. Black dashed line delineates YFP-PDAC. (C) Schematic representation of the chimera experimental approach. KPCY (*KRas*^*LSL-G12D/wt*^*; p53*^*F/F*^*; Pdx1-Cre; Rosa26-LSL-YFP*) embryonic stem cells were injected into C57BL/6J *Gt(ROSA)26Sor*^*tm9(CAG-tdTomato)Hze*^ mouse blastocysts to generate chimeras with a low contribution of YFP-expressing KPC cells in tdTomato+ healthy tissues. (D) Histological analysis of 4-month-old chimeric pancreas on serial sections. Black dashed line delineates the invasive front of the tumor. Black boxes in (B) and (D) indicate high-mag regions. All scale bars represent 100 μm.

The absence of lineage tracing observed in the PanIN-like lesions in the KFCkY model suggested that these lesions may be derived from wild-type tissue adjacent to the tumor. To test this hypothesis, we performed orthotopic injections of lineage-traced PDAC cells from *KRas*^*LSL-G12D/WT*^*; P53*^*F/F*^*; Pdx-Cre; Rosa26-LSL-YFP* (KPCY) mice. To exclude rare non-traced tumor cells, we sorted KPCY tumor cells for the YFP tracer before orthotopic transplantation (Figure 4A). Transplantation of KPCY cells led to efficient YFP-labeled PDAC formation in the host pancreas (Figure 4B). As in the KFCkY model, YFP-negative low-grade PanIN-like lesions positive for AB/PAS and Muc5AC were observed in close proximity to the tumor (Figure 4B). Thus, some low-grade PanIN-like lesions appear to be induced in host tissue as “bystanders” of PDAC growth.

To confirm the wild-type origin of the PanIN-like lesions, and to exclude the possibility that in vitro culture of primary PDAC tumor cells or orthotopic transplantation surgery could contribute to their induction, we performed PDAC/wild-type chimera experiments. Embryonic stem cells were generated from KPCY animals, and injected into host blastocysts ubiquitously expressing tdTomato (recognized by the RFP antibody). In the resulting chimeric mice, PDAC derived from the KPCY embryonic stem cells (ESCs) is YFP-labeled, whereas wild-type blastocyst-derived tissue is marked by tdTomato/RFP (Figure 4C). Histological analysis revealed YFP-positive PDAC, interspersed with RFP-positive stromal cells (Figure 4D). Importantly, we detected AB/PAS-positive low-grade PanIN-like lesions that were YFP-negative and RFP-positive, demonstrating their wild-type “bystander” origin (Figure 4D). These experiments confirm that PDAC tumors can induce PanIN-like lesions in the surrounding wild-type tissue.

### Tumors of Acinar and Ductal Origin Show Differences in AGR2 Expression

**Figure 5:**
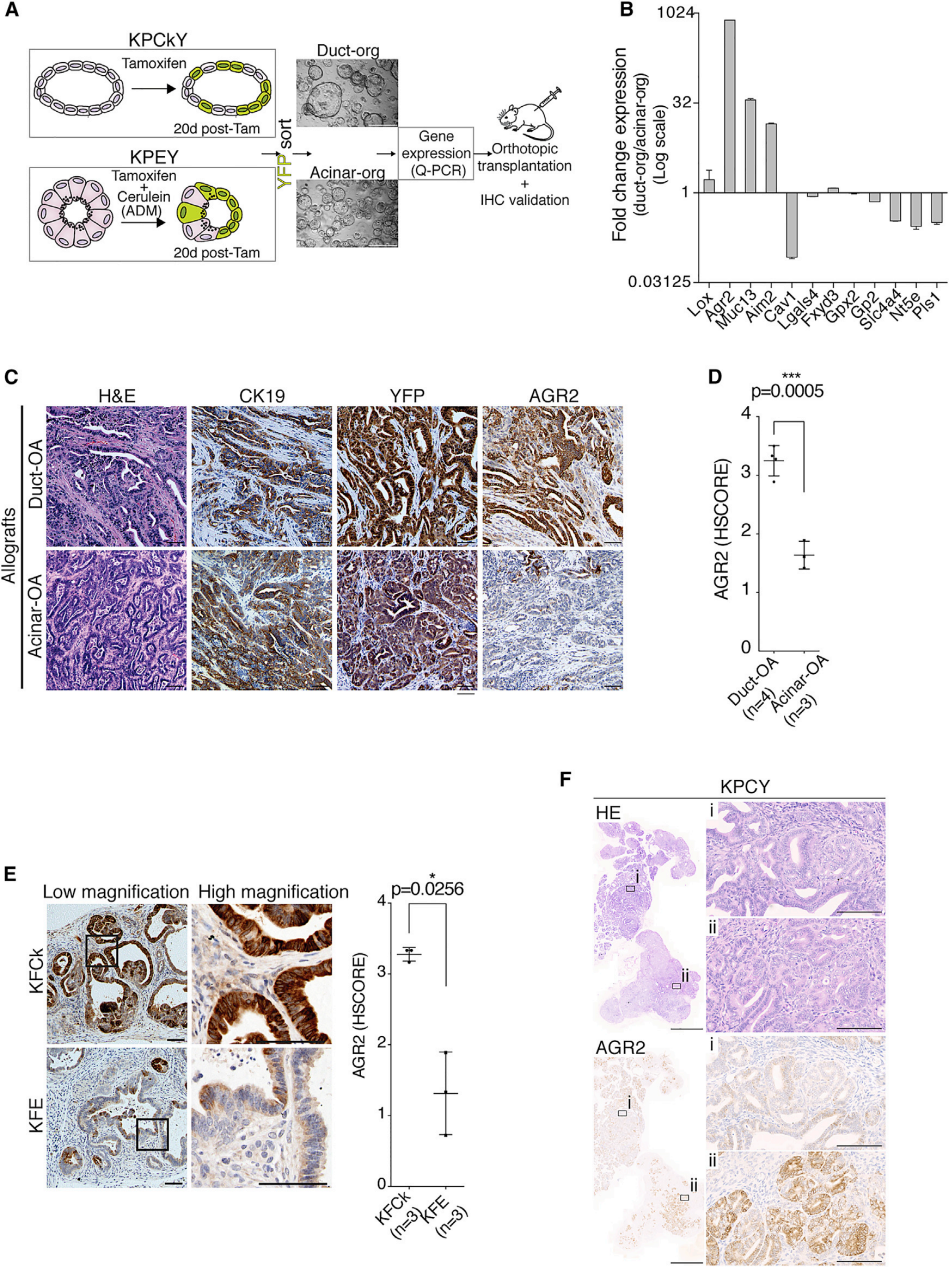
AGR2 Level and Extent of Expression Indicates Tumor Cell of Origin (A) Experimental approach to identify and validate potential cell of origin markers. Micrographs depict organoids used for gene expression analysis, derived from YFP-sorted cells from KPCkY and KPEY mice 20 days after in vivo recombination using tamoxifen (Tam). (B) Relative expression of selected genes used for PDAC classification in duct- and acinar-derived organoids, analyzed by qRT-PCR. Bar chart shows mean + SD. (C) Histological analysis of orthotopic allograft (OA) tumors generated from duct- (KPCkY) and acinar-derived (KPEY) organoids. (D) AGR2 blinded histological score (HScore) for four duct-OA tumors and three acinar-OA tumors, two sections per tumor. Dot plot shows mean ± SD. (E) AGR2 IHC stain of pancreatic pre-neoplastic lesions of three KFCk and three KFE animals, two sections per tumor, and respective HScore. Black boxes indicate high-mag regions. Dot plot shows mean ± SD. (F) H&E and IHC stain for AGR2 on PDAC from KPCY animals (*KRas*^*LSL-G12D/wt*^*; p53*^*F/F*^*; Pdx1-Cre; Rosa26-LSL-YFP*). Magnifications of AGR2 negative (i) and positive (ii) regions are shown. Scale bars represent 100 μm except for micrographs in (A) and in (F) (left panels), which are 500 μm and 2 mm, respectively. The p values were calculated using Welch’s t test. See also Figures S4 and S5.

The difficulty in distinguishing “true” PanINs from PanIN-like lesions has implications for tumor classification and prognosis, as the type of precursor lesion associated with the PDAC informs clinical decision-making (Cooper et al., 2013, Distler et al., 2014). Because some of the PanIN-like lesions identified at the time of tumor detection are reactive changes, not precursors, additional criteria are needed to aid PDAC stratification. To screen for potential markers indicative of tumor origin, we triggered oncogenic Ras activation and p53 inactivation in duct- and acinar-derived cells in parallel using KPCkY (*KRas*^*LSL-G12D/WT*^*; P53*^*F/F*^*; Ck19-Cre*^*ER*^*; Rosa26-LSL-YFP*) and KPEY (*KRas*^*LSL-G12D/WT*^*; P53*^*F/F*^*; Ela1-Cre*^*ERT2*^*; Rosa26-LSL-YFP*) mouse strains (Figure 5A). KPEY mice were also treated with cerulein to induce ADM. After 20 days, three-dimensional (3D) tumor organoids were established from YFP-positive cells from both models, and analyzed for the expression of a panel of genes described to stratify PDAC tumors into clinically distinct categories (Collisson et al., 2011) (Figure 5A). AGR2 (anterior gradient protein 2) showed the highest difference in mRNA level, being upregulated approximately 800-fold in duct-derived compared with acinar-derived tumor organoids (Figure 5B).

To determine whether AGR2 marks duct-derived PDAC, we transplanted duct- and acinar-derived KP organoids into the pancreas of NuNu mice to generate orthotopic allograft tumors (duct-OA and acinar-OA, respectively). Organoids from both origins generated YFP-traced invasive PDAC tumors embedded in dense YFP-negative fibrous stroma (Figure 5C), which were morphologically indistinguishable and consistent with the histology of KP pancreatic tumors (Bardeesy et al., 2006). However, duct-OA tumors showed widespread AGR2 staining, whereas acinar-OA tumors were low or negative for AGR2 (Figures 5C, 5D, S4A, and S4B). The differences in AGR2 expression were also observed by Q-PCR (Figure S4C).

To validate AGR2 as a marker of duct-derived PDAC, we analyzed AGR2 immunohistochemistry (IHC) staining in pancreata from KFCk and KFE mice (Figures 5E, S4A, and S4B). The extent of AGR2 staining and mRNA expression was significantly higher in duct-derived (KFCk) compared with acinar-derived (KFE) pancreatic lesions (Figures 5E and S4A–S4C).

Levels of AGR2 in PDAC inversely correlate with the extent of epithelial-to-mesenchymal transformation (EMT) of the tumor (Mizuuchi et al., 2015). In agreement with this finding, we observed that mesenchymal tumor regions did not express AGR2. To exclude EMT as a confounding factor in determining tumor origin, we restricted our analysis to the tubular, epithelial ductal cells. KPCY tumor cells that undergo EMT lose Ck19 expression (Figure S4D). Therefore, we assessed AGR2 expression in Ck19-expressing epithelial cells only. The difference between AGR2 levels in duct-derived and acinar-derived tumors was maintained (Figures S4E and S4F), suggesting that AGR2 truly distinguishes PDAC tumors of different origins.

Interestingly, KFCk mice showed significantly reduced survival compared with KFE mice (Figure S5A). Reduced survival was also observed in mice harboring duct-derived allografts compared with acinar-derived allografts (Figure S5B), supporting differential prognosis of PDAC of different origins.

Some of the most commonly used mouse models of PDAC, such as KPC (*KRas*^*LSL-G12D/WT*^*; P53*^*F/F*^*; Pdx1-Cre*), initiate oncogene expression at an embryonic stage, and the tumor cell of origin is unclear (Guerra and Barbacid, 2013). Interestingly, KPC PDAC tumors were heterogeneous for AGR2 staining, with large focal areas either strongly positive or negative for AGR2 (Figure 5F). Tumors of the same genotype derived from adult duct or acinar cells displayed either high or low AGR2 staining, in duct-derived or acinar-derived PDAC, respectively (Figure S4A). These observations are consistent with multifocal PDAC derived from both acinar and ductal cells in the KPC model (Maddipati and Stanger, 2015).

### Differences in AGR2 Expression in Human PDAC

**Figure 6:**
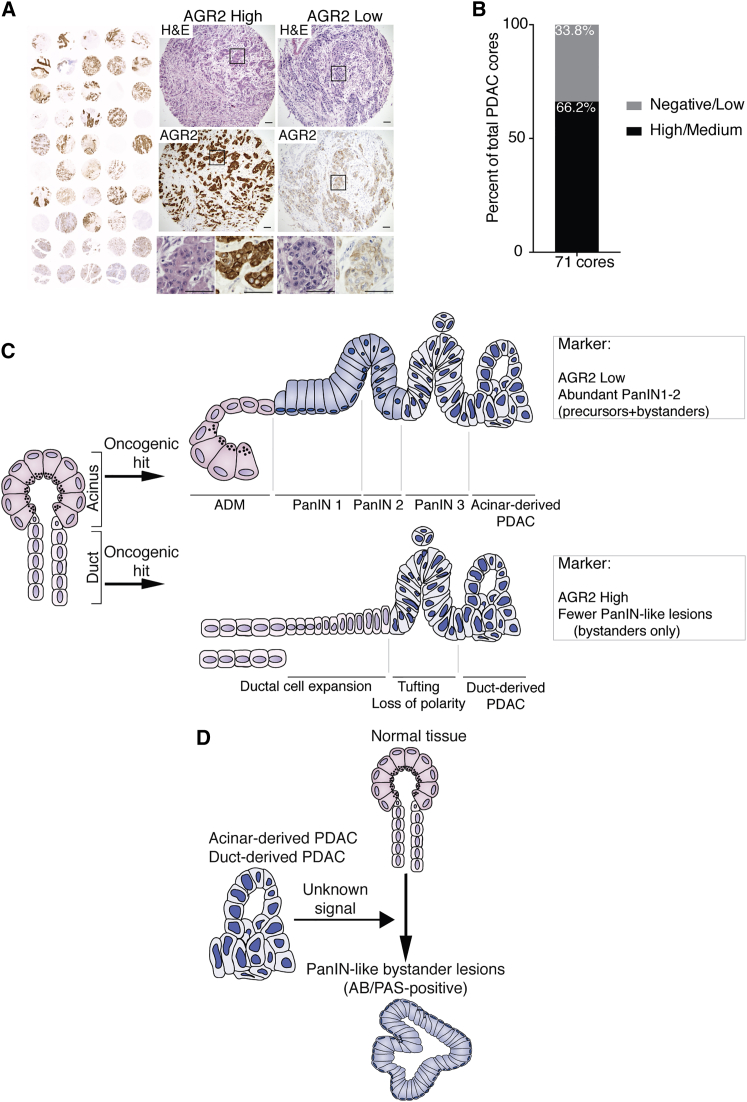
Human PDAC Tumors Show Distinct AGR2 Expression and Model Comparing Duct- and Acinar-Derived Tumors (A) Representative histological (H&E) and IHC analysis of AGR2 in human tumors on PDAC tissue microarray (Pa1002a, US Biomax), showing one AGR2 high core and one AGR2 low core. Black boxes indicate magnified regions shown below. (B) Quantification of AGR2 high/medium and low/negative PDAC tumor cores from two independent TMAs (Pa1001a and P1002a). (C) Schematic summary of duct-derived versus acinar-derived pancreatic tumor progression. Acinar cells respond to oncogenic hits by undergoing acinar-to-ductal metaplasia (ADM) that progresses to PanIN lesions (1–3) and culminates in PDAC. In contrast, duct cells harboring oncogenic mutations do not evolve into PanIN lesions but increase proliferation, leading to a crowding of the duct network and deformation of the ductal structures. Ductal transformation progresses to a stage resembling a non-mucinous carcinoma in situ that culminates in PDAC formation. Mature tumors from different origins are morphologically similar but can be distinguished by their levels and pattern of AGR2 expression and by the frequency of PanIN-like lesions. Duct cell-derived tumors are almost entirely AGR2-positive and present a reduced number of PanIN-like lesions, whereas acinar cell-derived PDAC only present focal AGR2-positive regions and show abundant low-grade PanINs. (D) Schematic representation of the origin of non-precursor PanIN-like lesions. The presence of developed PDAC induces the formation of PanIN-like bystander lesions originating from wild-type tissue. These are the only low-grade PanIN-like lesions found in duct-derived PDAC, but they are also present in PDAC from other cell origins. See also Figure S6.

Although proof of cellular origin in human PDAC is not yet possible, markers that stratify tumors according to origin in mice could help to refine the classification of PDAC in patients. Data from the human protein atlas (Uhlén et al., 2015) suggest that PDAC from different patients presents strongly divergent patterns and levels of AGR2 protein (Figure S6). To confirm this observation and control for differences in staining protocol, we performed AGR2 IHC stainings on two independent commercial tissue microarrays of human PDAC (US Biomax). We focused only on cuboidal epithelial cells in our analysis to exclude the effects of EMT on AGR2 levels (Mizuuchi et al., 2015). AGR2 staining patterns over 71 samples were highly divergent, with morphologically similar tumors expressing very high or very low levels of AGR2 (Figures 6A and 6B) as in our mouse models. These data are consistent with the existence of two classes of human PDAC tumors, which may be derived from acinar and duct cells. Together with the analysis of pre-neoplastic lesions, we speculate that analysis of AGR2 staining, specifically in epithelial cells of the tumor, could be of value in stratifying human PDAC.

## Discussion

Although studies of human PDAC originally put forward duct cells as cells of origin of the disease (Cubilla and Fitzgerald, 1976, Hruban et al., 2000b), work in GEM PDAC models proposed the acinar compartment as the principal contributor to PDAC development (Kopp et al., 2012). Here, we show that duct cells can initiate PDAC, giving rise to aggressive localized carcinoma in response to two oncogenic combinations: KRas^G12D^ together with inactivation of Fbw7 (KF model), or p53 (KP model). It is possible that duct cells are refractory to the genetic alterations studied previously. Conversely, Brg1 deletion has been found to promote neoplasia specifically in duct cells (von Figura et al., 2014). Alternatively, subtle differences in the CreER-driver lines employed (Ck19-CreER compared to HNF1β-CreER and Sox9-CreER used previously) may explain the altered duct cell susceptibility to transformation compared with previous studies.

Although it is well established that acinar cells develop PDAC in a PanIN-dependent manner (Guerra et al., 2007, Habbe et al., 2008) (Figure 6C), we demonstrate here that duct cells respond to the same oncogene activation by increasing proliferation, expansion toward the duct lumen, and progression to PDAC, independent of PanIN pre-neoplastic lesions (Figure 6C). Interestingly, the pre-neoplastic lesion associated with duct-derived *Ptf1a-Cre; KRas*^*G12D*^*; Brg1*^*f/f*^ tumors was IPMN (von Figura et al., 2014), whereas duct-derived precursor lesions in our models were exclusively non-mucinous. This suggests that, although the cell of origin is the principal factor determining the PanIN-dependent or independent course of tumor progression, it is likely that the genotype is a modulating factor in determining the course of duct-PDAC progression.

Despite the PanIN-independent progression, duct-derived PDAC, like the majority of human pancreatic tumors, exhibited PanIN-like lesions at the time of diagnosis. Our lineage-tracing studies, orthotopic allograft, and chimera results indicate that mucinous PanIN-like lesions can be divided into true precursor PanINs, which carry key oncogenic alterations, found from the earliest stages and exclusively in acinar-derived tumors (Figure 6C), and PanIN-like bystander lesions originating alongside fully developed tumors regardless of the cell of origin (Figure 6D). It is consensus in the clinic that high-grade PanIN lesions progress to PDAC, and thus their presence should be instructive of follow-up or surgery. However, the relevance of lower-grade lesions is less clear (Patra et al., 2017). Definitive diagnosis of pancreatic lesions in the context of malignant disease is challenging, and there is difficulty in distinguishing “reactive changes” of the epithelium from early PanINs (Hruban et al., 2001, Strobel et al., 2007), leading to possible inclusion of the latter in the PanIN classification and consequent misinterpretation of the type of PDAC. Although it has always been accepted that the proportion of early PanINs that progress to PDAC is low, our findings highlight the possibility that some lesions described as low-grade PanINs are in fact PanIN-like bystander lesions induced by the adjacent tumor.

These findings validate several long-standing clinical observations. Low-grade PanIN-like lesions are also found in the context of other pancreatic neoplasias such as pancreatic endocrine tumors, serous cystadenomas, solid pseudopapillary tumors, and ampullary tumors, which do not necessarily arise from PanINs and do not progress to PDAC (Recavarren et al., 2011, Stelow et al., 2006). Multicolor lineage tracing has demonstrated that malignant PanIN lesions are clonal entities (Maddipati and Stanger, 2015), expected to consist entirely of KRas mutant cells. However, a meta-analysis of low-grade PanIN lesions in human PDAC samples found that more than one-half do not harbor KRas mutations (Löhr et al., 2005). Using more sensitive sequencing techniques, a later study detected KRas mutations in most PanIN lesions, but often with mutation frequencies below 10% (Kanda et al., 2012), not compatible with a clonal origin. We speculate that these “PanINs” are in fact PanIN-like bystander lesions, arising in KRas wild-type tissue and possibly acquiring the mutation in one or two cells after lesion formation. Thus, data from human PDAC are consistent with the existence of PanIN-like bystander lesions as well as fully KRas mutant PDAC precursor PanINs. Because the only PanIN-like lesions occurring in murine duct-derived tumors are bystanders, these models will be invaluable in future research into these changes induced by the tumor in the surrounding tissue.

We have observed that PDAC originating in duct cells is associated with lower survival, both in the KP allografts and in the GEM models. Although additional studies have to be performed to assess the metastatic potential that is so common among pancreatic cancer patients, these data indicate that discriminating the origin of PDAC might inform patient prognosis. Because the classification of PDAC based on the type of pre-neoplastic lesion present at diagnosis is hampered by the presence of PanIN-like lesions, additional parameters will likely be required. We identified anterior gradient 2 (AGR2) as a putative marker of PDAC of ductal origin, and the marked differences in AGR2 expression pattern in PDAC patient samples, in conjunction with quantification of pre-neoplastic lesions, may aid the stratification of human PDAC tumors.

## Experimental Procedures

### Mouse Lines

*Ck19-Cre*^*ER*^ (Means et al., 2008), *Ela1-Cre*^*ERT2*^ (Desai et al., 2007), *Fbw7*^*F/F*^ (Jandke et al., 2011), *KRas*^*LSL-G12D/wt*^ (Jackson et al., 2001), *Nu/Nu* (Flanagan, 1966), *p53*^*F/F*^ (Marino et al., 2000), *Pdx1-Cre* (Hingorani et al., 2003), Rosa26-LSL-YFP (Srinivas et al., 2001), and C57BL/6J *Gt(ROSA)26Sor*^*tm9(CAG-tdTomato)Hze*^ (Madisen et al., 2010) mouse lines have been previously described. These lines were intercrossed to obtain the different genotypes in the study. All experiments performed in animals were approved by the Crick (formerly London Research Institute) Animal Ethics Committee and conformed to UK Home Office regulations under the Animals (Scientific Procedures) Act 1986 including Amendment Regulations 2012.

### Tamoxifen and Cerulein Injection

Cre expression was induced in adult (8-week-old) Ck19-Cre^ER^ and Ela1-Cre^ERT2^ mice by intraperitoneal injections of tamoxifen (Sigma) dissolved in peanut oil (Sigma) at a dose of 100 mg/kg of body weight. For KFCk, tamoxifen was injected once a day for 2 days, and for KFE, once a day for 5 days.

One week after tamoxifen treatment, KFE mice were intraperitoneally injected with cerulein (Sigma) dissolved in PBS, at a dose of 40 μg/kg body weight, three times a day on 3 days a week for 4 weeks, to induce chronic pancreatitis.

### Histopathology, IHC, and Immunofluorescence

Specimens were fixed overnight in 10% neutral buffered formalin, transferred to 70% ethanol 16 h later, and embedded in paraffin. For histopathology, paraffin-embedded pancreata were serially sectioned (3 μm) and every 20th section was stained with H&E.

For IHC, 4-μm sections were deparaffinized in Histo-Clear (National Diagnostics), followed by serial ethanol washes and water. Slides were subjected to heat-mediated antigen retrieval by incubation in 10 mM sodium citrate (pH 6.2) at 95°C for 15 min. Endogenous peroxidase was inactivated (except for IHC-immunofluorescence [IF]) with 1.6% H_2_O_2_ for 10 min, followed by epitope blocking for 1 hr at room temperature with 1% bovine serum albumin (Sigma-Aldrich), 5% normal donkey serum (EMD-Millipore), and 0.4% Triton X-100 (Sigma-Aldrich) diluted in PBS. All primary antibodies were incubated overnight at 4°C, followed by secondary incubation with biotin-conjugated antibodies for 45 min at room temperature or fluorescently conjugated antibodies for 2 hr. IHC signal was obtained with the avidin-biotin complex (ABC) solution (Vectastain ABC kit) and developed with peroxidase substrate kit 3,3′-diaminobenzidine (DAB) from Vector. IHC slides were counterstained with Mayer’s hematoxylin, and IHC-IF slides were incubated with DAPI. Antibodies used included the following: Ck19 (DSHB; Troma-III), GFP (used for YFP) (Abcam; ab6673), Muc5ac (Thermo Fisher; MS-145-B), HES1 (Chemicon/Millipore; AB5702), pH3 (Santa Cruz; sc-8656-R), pErk (p44/42; Cell Signaling; 4376), AGR2 (Atlas Antibodies; HPA007912), and RFP (Rockland/TEBU; 600-401-379).

### Alcian Blue/Periodic Acid-Schiff and Co-stain with GFP

To detect mucinous structures, alcian blue/periodic acid-Schiff (AB/PAS) staining was performed. Sections were deparaffinized by xylene treatment twice for 5 min. Sections were incubated twice in 100% industrial methylated spirits (IMS) and once in 70% IMS for 3 min each, followed by incubation with AB 1% (in 3% acetic acid) for 5 min. Slides were washed intensively in tap water and distilled water then incubated in 1% (w/v) periodic acid in distilled water for 5 min, washed again in distilled water, and incubated in Schiff (Fisher) for 15 min, and then a 10-min wash in running tap water. Nuclei were counterstained with Mayer’s hematoxylin for 1 min. The stain was differentiated in 1% acid alcohol for a few seconds (experimental slides performed in parallel) and washed in tap water for 5 min. Tissue was dehydrated in ethanol and cleared in xylene, and slides were mounted with coverslips using DPX mounting medium.

For the combination of AB/PAS with GFP staining, GFP immunostaining was performed first, followed by the AB/PAS staining procedure.

### KPCY Tumor Cell Isolation and Organoid Cultures

PDAC from KPCY (*KRas*^*LSL-G12D/WT*^*; P53*^*F/F*^*; Pdx-Cre; Rosa26-LSL-YFP*) animals was dissected, and cell dissociation was performed as described (Reichert et al., 2013) with a few modifications. The dissected tumor was immediately placed in 5 mL of cold G solution and kept on ice. Following mechanical dissociation, the tissue was transferred to a 50-mL Falcon with 15 mL of collagenase V (1 mg/mL in DMEM plus 1% [v/v] penicillin/streptomycin [10,000 U/mL with no FCS]) and incubated for 20 min in a water bath at 37°C. To inactivate the digestion, 15 mL of ice-cold G solution were added. The cell-dissociated PDAC was centrifuged at 300 × *g* for 5 min with low deceleration, the supernatant was discarded, and the pellet incubated in trypsin-EDTA for 5 min at room temperature. To stop the reaction, 2 mL of soybean trypsin inhibitor were added, followed by 15 mL of ice-cold G solution and centrifugation for 5 min at 300 × *g*. The pellet was resuspended in 10 mL of PBS with 2% (v/v) FCS and passed through a 70-μm filter. Cells were plated in growth factor-reduced Matrigel to grow as 3D organoid cultures as described (Huch et al., 2013).

### Generation of Duct- and Acinar-Derived Organoids

KPCkY (*KRas*^*LSL-G12D/WT*^*; P53*^*F/F*^*; Ck19-Cre*^*ER*^*; Rosa26-LSL-YFP*) and KPEY (*KRas*^*LSL-G12D/WT*^*; P53*^*F/F*^*; Ela1-Cre*^*ERT2*^*; Rosa26-LSL-YFP*) 8-week old animals were intraperitoneally injected with tamoxifen, as above. KPEY mice were then subjected to 1 week of cerulein-induced chronic pancreatitis to induce in vivo ADM. Animals were sacrificed 20 days post-tamoxifen, and tumor cells were isolated as described above. YFP-positive single cells (duct-derived KPCkY and acinar-derived KPEY) sorted by fluorescence-activated cell sorting (FACS) were grown as 3D organoids in growth factor-reduced Matrigel.

### Orthotopic Transplantation

Duct- and acinar-derived organoids were dissociated into single cells by trypsin treatment, and YFP-positive cells were isolated by FACS. The peritoneum of anaesthetized *Nu/Nu* mice was surgically opened, and 150 × 10^3^ tumor cells suspended in growth factor-reduced Matrigel (50 μL) were injected in the pancreatic tail. The peritoneal wall was sutured with absorbable sutures, and the skin was closed with clips. A 0.05 mg/kg dose of Vetergesic was subcutaneously injected immediately after surgery. Tumors were collected 20 days post-transplantation for the detection of PanIN-like bystander lesions. For acinar and duct-derived allografts (acinar-OA and Duct-OA), *Nu/Nu* mice were allowed to develop tumors up to 1 mm^3^.

### KPCY ESCs and Generation of Chimeras

ESCs were generated as described (Czechanski et al., 2014) with minor modifications. ESCs were obtained from KPCY mouse blastocysts and derived in serum free (Gibco knockout serum replacement and DMEM) conditions in the presence of LIF and PD 0325901 (MEK inhibitor). Following two to three passages, ESCs were placed in standard serum medium (15% plus LIF) and maintained in standard murine ESC (mESC) conditions. Chimera production was performed following previously published guidelines (Behringer et al., 2003). KPCY ESCs were injected into C57BL/6J *Gt(ROSA)26Sor*^*tm9(CAG-tdTomato)Hze*^ mouse blastocysts. Tumors of the KPCY/Tomato chimeric mice were detected at 4 months of age.

### AGR2 HScore

AGR2 scoring was performed by six independent observers as a blinded analysis. IHC staining intensities were scored for the duct-like structures in each tumor from 0 (no staining) to 4 (high and widespread staining). Mean average score for each tumor of each genotype was calculated. At least three mice per genotype and two tissue sections per mouse were used.

### AGR2 on Tissue Microarrays

Two independent tissue microarrays (TMAs) were acquired from US Biomax (PA1001a and PA1002a). IHC for AGR2 was performed as above. Cores were excluded if absent in the slide or if repeated between the TMAs. Two additional cores were excluded due to differences in classification (high AGR2 expression in one TMA and medium expression in the other). A total of 71 cores were used for analysis.

### Quantitative RT-PCR

RNA was isolated from dissociated organoids or paraffin-embedded tumors using the RNeasy mini-kit (QIAGEN) or the RecoverAll Total Nucleic Acid Isolation Kit (Ambion, Life Technologies), respectively. cDNA was generated using the Transcriptor First Strand cDNA synthesis kit (Roche Diagnostics) and was used for qPCR SYBR-Green (Life Technologies) detection using the primers listed in Supplemental Experimental Procedures.

### Statistics

Data were analyzed using GraphPad Prism software (GraphPad Software) and presented as mean ± SD. Statistical analysis was performed using two-sided Welch’s t test (with correction for unequal variance) for bar charts or dot plots. A value of p ≦ 0.05 was considered statistically significant. The exact p value is given for each experiment.

## Author Contributions

R.M.M.F. conceived and designed the study, performed most experiments, analyzed and interpreted data, and drafted the manuscript. R.S. assisted with study design and interpretation of data. H.A.M. assisted with study design and interpretation of data. E.N., B.S.-D., and R.K.S. assisted with IHC stainings and histological analysis. G.S. and A.Q. assisted with lesion identification and description. I.R. generated KPCY EC cells and KPCY/TdTomato chimeras. A.B. conceived and designed the study, supervised experiments, and drafted the manuscript.

## Supplemental Information

Document S1. Supplemental Experimental Procedures and Figures S1–S6

Document S2. Article plus Supplemental Information
