# Supplementary material for: Duct- and Acinar-Derived Pancreatic Ductal Adenocarcinomas Show Distinct Tumor Progression and Marker Expression
Source: Cell Rep. 2017 Oct 24;21(4):966–78. doi: 10.1016/j.celrep.2017.09.093 (PMC5668631; doi:10.1016/j.celrep.2017.09.093)
Supplement: Document S1. Supplemental Experimental Procedures and Figures S1–S6 [file mmc1.pdf]

Cell Reports, Volume *21*

Supplemental Information

Duct- and Acinar-Derived Pancreatic Ductal Adenocarcinomas Show Distinct Tumor Progression and Marker Expression

Rute M.M. Ferreira, Rocio Sancho, Hendrik A. Messal, Emma Nye, Bradley Spencer-Dene, Richard K. Stone, Gordon Stamp, Ian Rosewell, Alberto Quaglia, and Axel Behrens

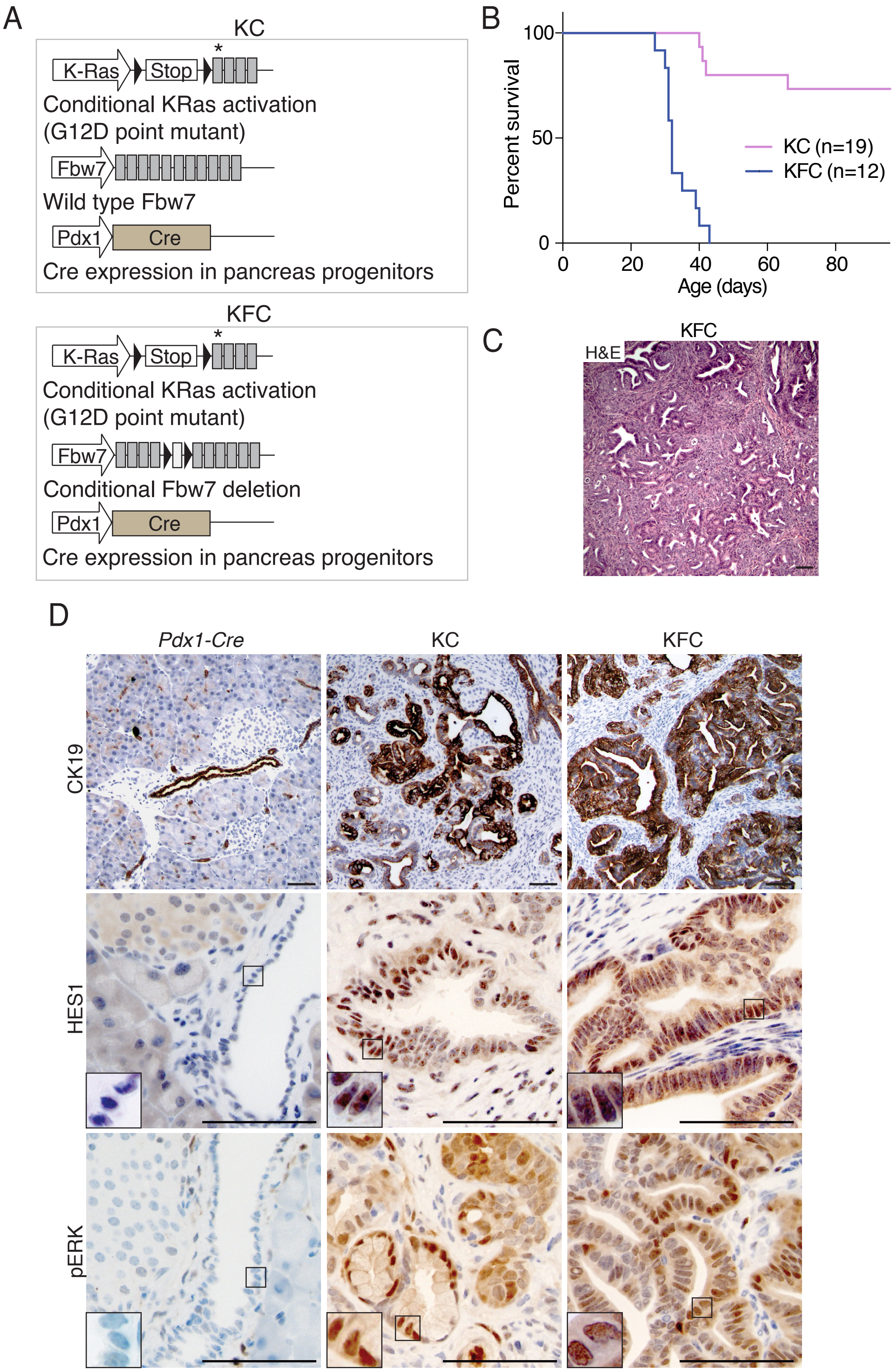

Figure S1, related to Figure 1: Immunohistological comparison between KC and KFC mouse models

(A) Schematic representation of the KC (*KRas*$^{LSL\text{-}G12D/wt}$; *Pdx1-Cre*) and KFC (*KRas*$^{LSL\text{-}G12D/wt}$; *Fbw7*$^{F/F}$; *Pdx1-Cre*) mouse models. Cre expression is driven by the *Pdx1* promoter. Black triangles indicate loxP sites flanking either exon 5 of the *Fbw7* gene or a STOP cassette preceding exon 1 of the *KRas* gene. Asterisk indicates G12D point mutation. (B) Kaplan-Meier curve comparing survival of 19 KC mice (red) and 12 KFC mice (blue). (C) Representative hematoxylin and eosin (H&E) stain of PDAC from a 4-week-old KFC mouse. (D) Representative immunohistological analysis of Cytokeratin 19 (CK19), HES1 and phosphorylated ERK (pERK) in *Pdx1-Cre* (control) pancreas and KC and KFC pancreata. Scale bars represent 100µm.

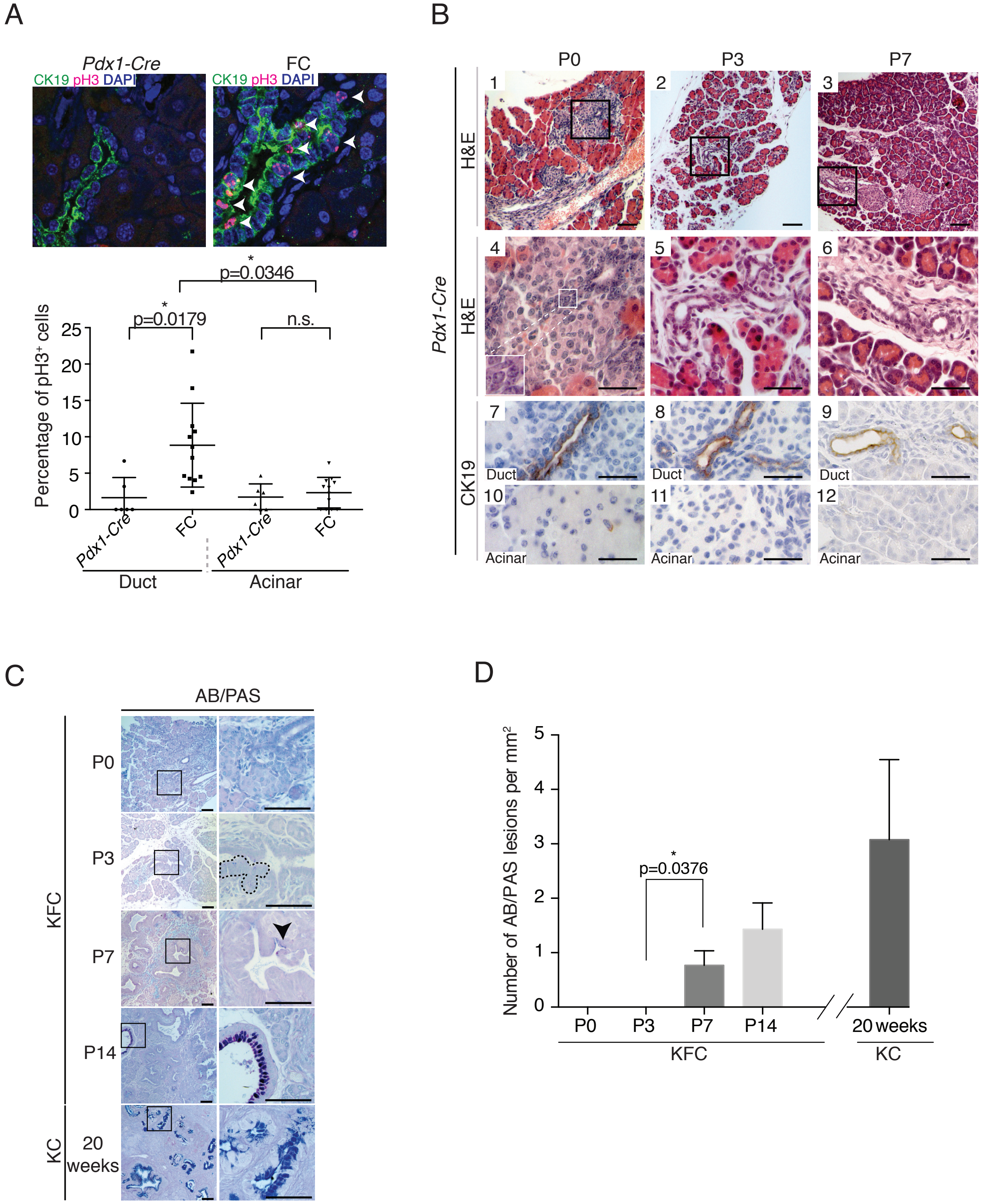

Figure S2, related to Figure 1: KFC models display ductal cell overproliferation and early ductal dysplasia
(A) Immunofluorescence staining of CK19 (green) and phosphorylated histone 3 (pH3) (red) in age-matched adult pancreas of *Pdx1-Cre* control and *Fbw7*$^{F/F}$; *Pdx1-Cre* (FC) mice and quantification of pH3^{+} cells in acinar or ductal compartments. White arrowheads indicate pH3^{+} ductal cells. Graph shows mean values ±SD. Datapoints indicate individual sections. *P* values were calculated using Welch's t-test, n=3 mice per genotype. (B) Time-course analysis of early postnatal pancreatic development in *Pdx1-Cre* control used to distinguish normal postnatal changes in pancreatic morphogenesis from the oncogene-related alterations in the KFC model in Figure 1D. (1-3) H&E low magnification images. Black boxes indicate area magnified in images 4-6. White box highlights absence of mitotic figures in normal (*Pdx1-Cre*) ductal cells at P0. (7-12) Ck19 immunohistochemical analysis of ductal cells (7-9) and acinar cells (10-12). For each time point two litters, each comprising more than 3 *Pdx1-Cre* mice, were analyzed. Scale bars in panels 1-3 represent 100 µm. Scale bars in panels 4-12 represent 50 µm. (C) AB/PAS staining of KFC pancreas at P0, P3, P7 and P14. KC mouse pancreas 20 weeks after birth was used as a positive control for AB/PAS staining of PanIN lesions. Black boxes indicate area magnified in images on the right. Dotted lines indicate ductal transformation with no AB/PAS staining. Black arrowhead indicates AB/PAS positive regions. Scale bars represent 100 µm. (D) Number of AB/PAS positive lesions per mm^2 of pancreatic tissue. 2 mice per time point were used and 3 tissue sections were stained per mouse. Bar chart shows the mean values. Error bars indicate standard deviation (SD). Statistical significance was tested with Welch's t-test. *P* values are indicated.

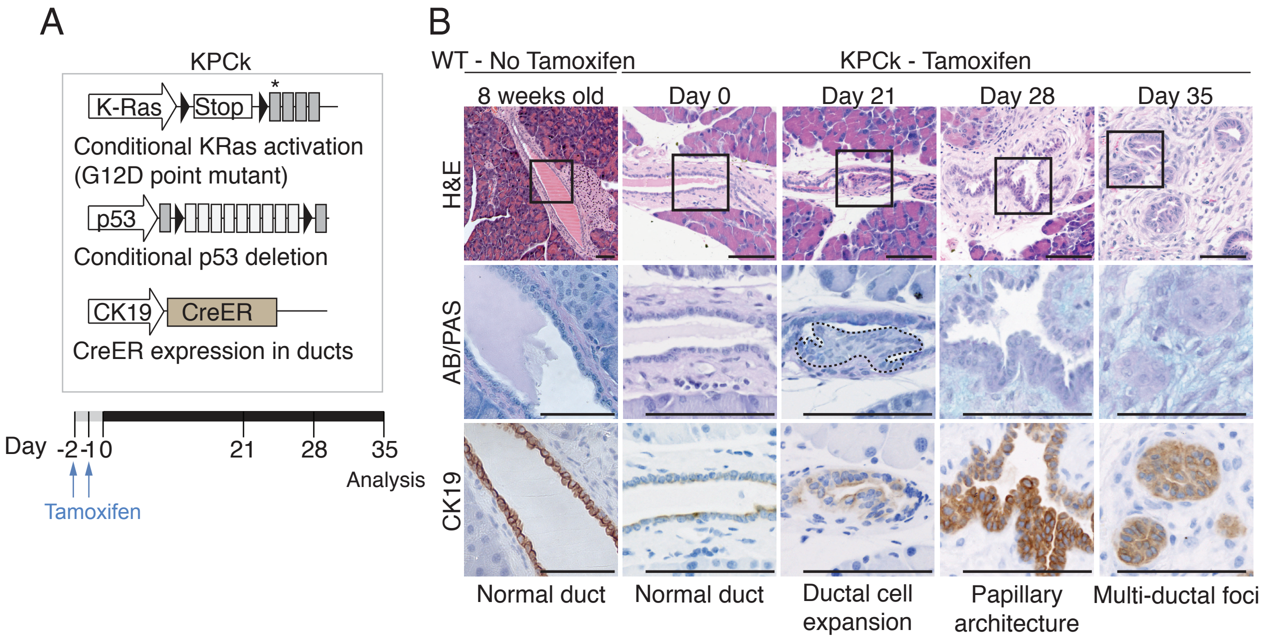

Figure S3, related to Figure 2: p53 loss together with $KRas^{G12D}$ expression in ductal cells does not induce formation of low-grade PanINs at early stages of tumor development

(A) Schematic representation of the KPCk ($KRas^{LSL\text{-}G12D/wt}$; $p53^{F/F}$; $Ck19\text{-}Cre^{ER}$) mouse and time-course experimental approach. Black triangles indicate loxP sites; asterisk indicates the exon with the G12D mutation. 8-week-old mice were intraperitoneally injected with tamoxifen (100 mg/kg of body weight) once a day for two days. Pancreatic tissue was collected for analysis at day 0, 21, 28 and 35.

(B) Immunohistological analysis of wild type (WT) 8-week-old pancreas and time-course analysis of ductal transformation in the KPCk model. Regions of interest marked in low-magnification H&E images are analyzed for AB/PAS staining and Ck19 immunological staining below. At least 3 mice were used per time point. Black dashed line highlights ductal expansion. All scale bars represent 100 μm.

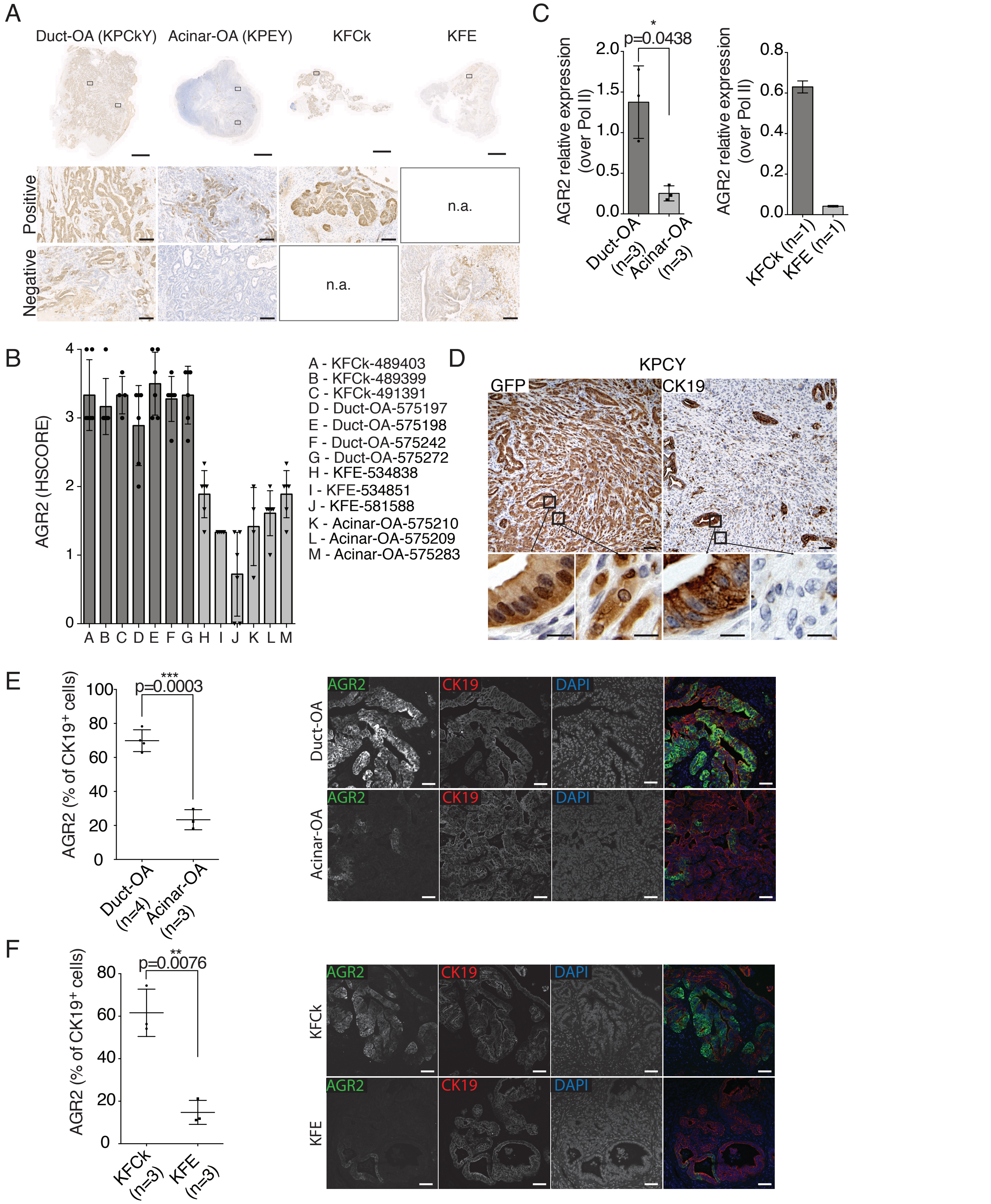

Figure S4, related to Figure 5: AGR2 level and extent of expression is indicative of tumor cell of origin in mice

(A) AGR2 IHC stains of acinar and duct-derived KP organoid allograft tumors (acinar-OA and duct-OA), and KFE and KFCk tumors. Top row shows whole-pancreas sections (Scale bars: 2 mm). Positive and negative regions for AGR2 are magnified (Scale bars: 100 µm). n.a., not applicable. (B) AGR2 HScore for 3 KFCk tumors, 3 KFE tumors, 4 Duct-OA allografts, and 3 acinar-OA allografts, 2 sections per tumor. AGR2 staining was blind-scored from 0 (no staining) to 4 (high, widespread protein stain) by 6 independent observers. Bar chart shows the mean values for each tumor ±SD. Dots indicate individual observer scores. (C) AGR2 expression levels tested by RT Q-PCR on RNA extracted from paraffin-embedded tumors. Expression levels are plotted relative to Pol II expression. (D) Immunohistochemical GFP and CK19 analysis of PDAC from the KPCY model. Magnified areas demonstrate that cuboidal/columnar GFP^+ tumor cells are positive for CK19 but spindle-like GFP^+ tumor cells lose CK19 expression. (E) Co-immunofluorescence stain for CK19 and AGR2 on paraffin-embedded Duct-OA and Acinar-OA tumors. Percentage of CK19-positive cells that expressed AGR2 is quantified on the left. Dot plot shows the mean values for each mouse ± SD. n=3 mice for Acinar-OA and n=4 mice for Duct-OA. (F) Immunofluorescence stain for CK19 and AGR2 on paraffin-embedded KFCk and KFE tumors. Percentage of CK19-positive cells that expressed AGR2 is quantified on the left. Dot plot shows the mean values of each mouse ± SD. n=3 mice per tumor type. Statistical significance was tested with Welch's t-test. *P* values are indicated. Scale bars in (A) represent 100 µm, in (D) represent 100 µm for low magnification and 20 µm for high magnification, and in (E) and (F) represent 50 µm.

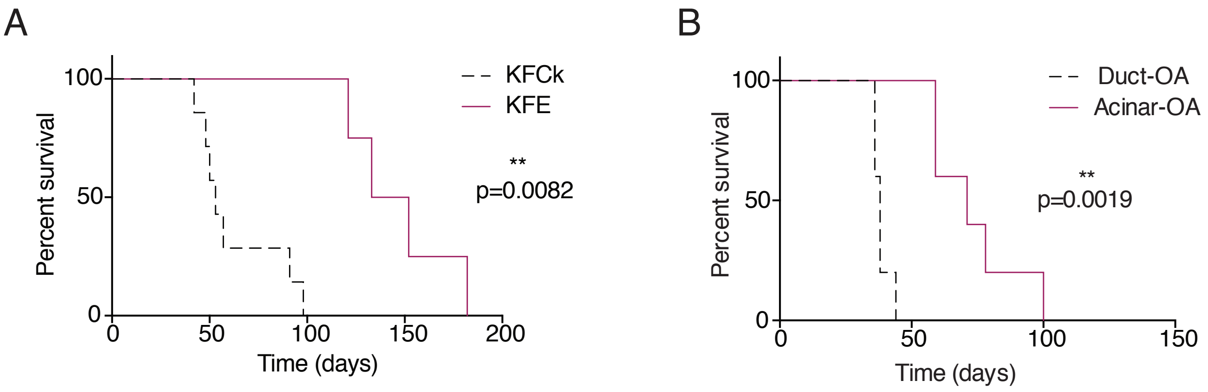

Figure S5, related to Figure 5: Survival analysis of duct-derived and acinar-derived PDAC tumor models
(A) Kaplan-Meier survival curve for duct-specific and acinar-specific PDAC GEMs, KFCk and KFE respectively. n=7 for KFCk and n=4 for KFE. (B) Kaplan-Meier survival curve for Duct-OA and Acinar-OA tumor mice. n=5 mice for both Duct-OA and Acinar-OA. OA, orthotopic allograft tumor from KPCkY (duct-derived) or KPEY (acinar-derived) organoids. Statistical significance was tested with the Mantel-Cox (Log-rank) test. *P* values are indicated.

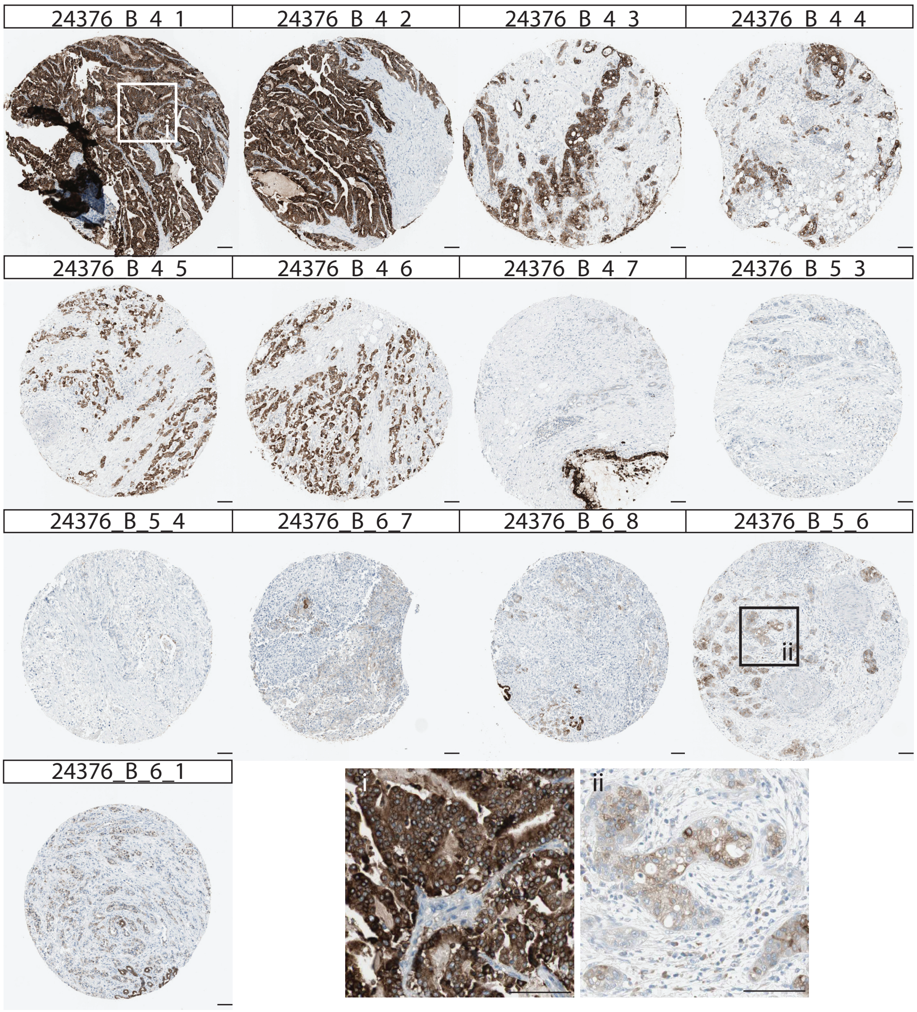

Figure S6, related to Figure 6: AGR2 level and extent of expression might be indicative of tumor cell of origin in humans

AGR2 IHC of human PDAC cores on the Human Protein Atlas (www.proteinatlas.org). i and ii indicate examples of high and low staining respectively. Scale bars represent 100 µm.

Supplemental Experimental Procedures

Primers used in this study:

Gene	5'-Fw primer-3'	5'-Rv primer-3'
Lox	ctcctgggagtggcacag	cttgctttgtggccttcag
AGR2	cctcaacctggtctatgaaaca	cgtcagggatgggtctacaa
Muc13	cccacaggttcctttttgtg	ccgttatccatctttaagcactg
Aim2	tgggctgtttaaagtccagaa	cacctccattgtccctgttt
Cav1	ccagggaaacctcctcaga	ccggatgggaacagtgtaga
Lgals4	catgcctgagcactacaagg	cgaggaagttgatggactgaa
Fxyd3	tcagacagaaacccagtcacc	ggtccatcttcagcagttgtg
Gpx2	gttctcggcttcccttgc	ttcaggatctcctcgttctga
Gp2	acaggcatgaccctgtgaa	ttgatggtggaatcacgttg
Slc4a4	actgtctccagtgcaagtagga	tgtcagattcctgtgggtca
Nt5e	atgaacatcctgggctacga	gtccttccacaccgttatcaa
Pls1	cattgatctcaacttactggaagg	catccagttgcggaatgtc
Pol II	aatccgcatcatgaacagtg	tcatccattttatccaccacct
